# Assessment of Health-Promoting Lifestyle among Dental Students in Zagreb, Croatia

**DOI:** 10.3390/dj9030028

**Published:** 2021-03-06

**Authors:** Larisa Musić, Tonći Mašina, Ivan Puhar, Laura Plančak, Valentina Kostrić, Mihaela Kobale, Ana Badovinac

**Affiliations:** 1 Department of Periodontology, School of Dental Medicine, University of Zagreb, HR-10000 Zagreb, Croatia; lmusic@sfzg.hr (L.M.); puhar@sfzg.hr (I.P.); 2 Department of Physical Education, School of Medicine Zagreb, University of Zagreb, HR-10000 Zagreb, Croatia; tonci.masina@mef.hr; 3 School of Dental Medicine, University of Zagreb, HR-10000 Zagreb, Croatia; lplancak@sfzg.hr; 4 Private Dental Practice, HR-10000 Zagreb, Croatia; kostricvalentina@gmail.com (V.K.); kobale.mihaela@gmail.com (M.K.)

**Keywords:** dental education, dental students, healthy lifestyle, surveys and questionnaires, health behavior, health promotion, school health services

## Abstract

As future healthcare professionals, dental medicine students are expected to exhibit healthy lifestyle behaviors. This study aims to assess the health-promoting behaviors among undergraduate dental medicine students of all six academic study years at the University of Zagreb, and determine their predictors. Students were invited to complete a two-part survey, consisting of a self-reported sociodemographic questionnaire and the Health-Promoting Lifestyle Profile II (HPLP II). Three hundred and forty-nine students completed the survey; the response rate was 60.3%. The total mean HPLP II score was 2.64 ± 0.34. Students in the second academic study year scored the lowest (2.50 ± 0.33), and students in the sixth academic study year scored the highest (2.77 ± 0.32). Health responsibility was the overall lowest scored subcategory, while interpersonal relations was scored the highest. Female students reported lower spiritual growth and stress management than male students. Higher body mass index (BMI) was related to lower health responsibility. Smoking, place of residence and the age of participants did not seem to have an impact on health-promoting behaviors. Dental students at our faculty exhibit moderate health-promoting behaviors, even in the absence of a formal health-promoting course in the existing curriculum.

## 1. Introduction

As defined by the World Health Organization, a healthy lifestyle entails living in a manner that lowers the risk of severe illness or early death [1]. Behavioral risk factors such as smoking, poor nutrition, and physical inactivity increase the risk of chronic, non-communicable diseases, which cumulatively led to an estimated 71% of all deaths globally, as reported by the latest available data from the year 2016 [2]. As these risk factors are modifiable, through their control, many non-communicable diseases may be avoided, if not prevented [3].

Indeed, a longitudinal US population-based study including more than 120,000 subjects reported that people who exhibited low-risk lifestyle habits (e.g., non-smoking, healthy weight (BMI < 25 kg/m^2^), ≥30 min/day of moderate to vigorous physical activity, moderate alcohol intake, and a high diet quality score) enjoyed significantly longer lives than those who exhibited none [4]. 

Poor health behaviors and habits, such as poor nutrition and, consequently, obesity [5,6], physical activity, and sedentary behavior [7], seem likely to persist into a person’s adulthood. Thus, while healthy lifestyle behaviors are essential in all periods of life, the adoption of correct habits and attitudes is preferable during youth and adolescence [8,9]. 

The beginning of university education represents a significant turning point in an individual’s life [10]. A large body of evidence suggests that in this period, many students face challenges in the maintenance of and adherence to healthy lifestyle behaviors, in terms of physical activity [11,12], nutrition, substance use [13,14], social connections, personal behaviors [15], and sleeping habits [16,17]. 

Health promotion and health-promoting behaviors are complementary to a healthy lifestyle and include actions, attitudes, and beliefs which individuals enforce to stay healthy, maintain good physical fitness, prevent disease. They also enable individuals to increase control over and to improve their health [1,18].

Students of biomedical disciplines are expected to grow into the role of health behavior promoters. To facilitate this growth, an educational framework should be set in order to raise awareness, educate, and motivate students to adopt and maintain a healthy lifestyle. In the curriculum of the integrated study program at our faculty, however, there is no course dedicated to this subject. In 2018, an interdisciplinary promotion-preventive initiative under the name of “Healthy University” was founded at the University of Zagreb. 

A number of studies have assessed health-promoting behaviors in student populations around the world [19,20,21,22,23,24] using the Health-Promoting Lifestyle Profile II (HPLP II), first described by Walker et al. [18]. In Croatia, health-promoting lifestyles have previously been assessed among medical students [25,26]. However, such data for the population of dental students is missing. 

Thus, we conducted the present study with the primary objective of evaluating the health-promoting behaviors among students of dental medicine in Croatia and observing possible differences related to the year of their academic study. Furthermore, the secondary objective was to elucidate the possible predictors of such behaviors. The findings of this study may be implemented in tailored programs and activities, aiming to improve and promote healthy lifestyles.

## 2. Materials and Methods

This cross-sectional study was conducted on the population of undergraduate students of the School of Dental Medicine, University of Zagreb, during February and March 2019. The Ethics Committee of the School of Dental Medicine approved the study (No: 05-PA-30-11/2018). The study was performed in accordance with the Declaration of Helsinki. 

The Croatian version of the HPLP II questionnaire was prepared in Google Form and shared for online access. The questionnaire was anonymous and voluntary. Participant information was provided in written form before students moved forward with the survey. They were informed about the type and the purpose of the survey and the ability for withdrawal at any point in time. 

The survey consisted of two parts. The first part was a self-reported questionnaire on seven sociodemographic items (age, gender, weight, height, year of study, place of residence, and smoking status). The body mass index (BMI) of participants was calculated [27]. The second part of the survey was a Croatian version of the HPLP II questionnaire examining attitudes toward health, healthy lifestyles, and the psychosocial aspects of respondents, previously translated by Mašina et al. [25]. The HPLP II questionnaire consists of 52 questions divided into two main categories and six subcategories. The category of positive health behavior includes the following subcategories: health responsibility, physical activity, and nutritional habits, and the psychosocial value category includes spiritual growth, interpersonal relations, and stress management. Health responsibility covers nine questions about an individual’s attitude to their health. The physical activity subcategory has eight questions related to regular exercise and activity. Nine questions in the subcategory nutritional habits refer to the choice of meal-type and nutritional value. The subcategories of spiritual growth and interpersonal relationships also have nine questions related to the relationships with oneself and others. Stress management has eight questions that include stress recognition and actions to control it. Possible answers follow a four-point scale ranging from 1 (never) to 4 (routinely). The total HPLP II score and scores of the individual subcategories of the HPLP II questionnaire were calculated according to the available scoring instructions [18], by calculating the mean of the individual’s responses. A higher HPLP II score indicates a better health-promoting lifestyle.

The value of the Cronbach alpha coefficient of the Croatian version of HPLP II questionnaire completed by the students of Dental Medicine was 0.91, and ranged from 0.66 to 0.84 for subcategories. 

Data Analysis

The data were organized into a Microsoft Excel spreadsheet (Microsoft Inc., Redmond, WA, USA), and the statistical analyses were performed using IBM SPSS^®^ Statistics version 25.0 for Windows (IBM Corp., Armonk, NY, USA). The normality of the distribution for continuous variables was analyzed by the Kolmogorov–Smirnov test, and for further statistical analyses, parametric tests were used. Differences between the years of study, with respect to the value of individual domains of the HPLP II questionnaire, as well as the total value of the HPLP II questionnaire, were analyzed by one-way ANOVA and post hoc test. Multiple regression analysis was used to examine predictors for the overall HPLP II score and for the scores of six health-promoting lifestyle subscales. The significance level was set at 5%. 

## 3. Results

### 3.1. Demographic Data

A total of 578 students from year 1 to 6 were enrolled in the academic program in the year 2018/2019 at the School of Dental Medicine, University of Zagreb. Three hundred and forty-nine of these students took part and completed the survey, with the response rate being 60.3%. Among those who completed the survey, 14.6% of the participants were male, and 85.4% were female, and the mean age of the students was 22.3 ± 1.03. The percentage of male students was lowest in the fifth year (7.0%) and highest in the second year (21.2%). The response rate of the students was highest in the first year (71.8%), while the lowest response rate was in the fifth year (54.8%). The distribution of the students by academic year, gender, and age, as well as the response rate, are shown in Table 1.

Among those who completed the survey, 37.0% of the participants lived in their family home, 16.9% lived in a student dormitory, 28.4% lived in a rented apartment, and 17.8% lived elsewhere. Furthermore, the majority of the participants were non-smokers (80.5%), 13.5% of the participants were smokers, and the remaining 6.0% were former smokers (Table 2).

### 3.2. HPLP II Overall Score and Subcategories Score by Academic year

The overall mean HPLP II score was 2.64 ± 0.34, ranging 1.73–3.50. The sixth-year students had the highest scores of 2.77 ± 0.32, while second-year students showed the lowest scores of 2.50 ± 0.33 (*p* < 0.001). When analyzing the total mean scores of the subcategories of the HPLP II questionnaire, the students scored the highest score on interpersonal relations (3.30 ± 0.39), followed by spiritual growth (2.95 ± 0.47), nutrition (2.72 ± 0.41), stress management (2.37 ± 0.46), and physical activity (2.23 ± 0.65), while scoring the lowest in the subcategory of health responsibility (2.17 ± 0.57).

The results of the mean scores in the HPLP II subcategories showed that sixth-year students achieved higher values than their colleagues in lower years of study (first to fifth) in the subcategories of physical activity (2.46 ± 0.66), nutrition (2.85 ± 0.46), interpersonal relations (3.42 ± 0.31), stress management (2.53 ± 0.41), and spiritual growth (3.06 ± 0.50), and the fifth-year students achieved the highest values (2.27 ± 0.51) in the domain of health responsibility. Second-year students achieved the worst results in all subcategories compared to students in other years of study (Table 3). 

One-way ANOVA assessed that differences in overall HPLP II mean score according to the year of study were statistically significant (*p* = 0.001). Likewise, significant differences were observed according to the year of study for the mean scores of HPLP II subcategories physical activity (*p* = 0.006), nutrition (*p* = 0.002), and stress management (*p* = 0.001). In order to establish the differences between the year of study, and the statistically significantly different subcategories (physical activity, nutrition, and stress management), as well as the overall HPLP II mean scores, a post hoc test was carried out. The results of one-way ANOVA and post hoc comparisons are shown in Table 3. 

Multiple regression analysis of the demographic variables (age, gender, BMI, year of study, place of residence, and smoking habit) with the overall HPLP II score and score of six subcategories was performed to determine the predictors of a healthy lifestyle in the participants. A higher year of study was shown to be a predictor for a higher total score of HPLP II (β = 0.207, *p* < 0.001). In the subcategory of health responsibility, higher BMI was related to lower health responsibility (β = −0.149, *p* = 0.009), and a higher year of study was a predictor for better health responsibility (β = 0.108, *p* = 0.049). Higher BMI and higher year of study were predictors for better nutrition (β = 0.124, *p* = 0.028 and β = 0.177, *p* = 0.001, respectively), while age was shown to be a predictor for higher spiritual growth and female gender for lower spiritual growth (β = 0.113, *p* = 0.040 and β = −0.137, *p* = 0.016, respectively). A higher year of study was shown to be a predictor for better stress management (β = 0.218, *p* < 0.001), and female students seemed to have weaker stress management than male students (β = −0.171, *p* = 0.002) (Table 4). No predictors could be found for the subcategories of interpersonal relations and physical activity, and demographic variables smoking and place of residence did not show any significant effect on the health-promoting lifestyle (data not shown). Generally, the multiple regression analysis that was performed to determine predictors of a healthy lifestyle explained only 5–8% of the variance.

## 4. Discussion

This study has assessed, for the first time, the health-promoting behavior of dental medicine students in Croatia. The overall mean HPLP II scores (2.64 ± 0.34) among dental students at the School of Dental Medicine in Zagreb highlights their moderate health-promoting lifestyle. The results of our study are consistent with previously reported data on a Croatian population of medical students [25]. Moreover, similar findings were highlighted by other international studies conducted on a population of dental students (2.49 ± 0.32) [20], medical students [19], nursing students [28], and mixed faculty students [22].

When assessing the results according to the year of the academic study, the sixth-year students scored the highest results, whereas the second-year students engaged least frequently in health-promoting behavior. Moreover, the year of study was highlighted as a predictor for a higher total score of HPLP II. As of yet, there is no official educational health-promoting framework at our faculty. Thus, we can only speculate that sixth-year students’ awareness and motivation for health-promoting behavior is progressively stimulated and grown throughout the course of their healthcare education. The lowest scores of the second-year students were closely followed by the first-year students, with no statistically significant difference in scores between the two. While there is no clear explanation for this observation, it could be attributed to the high academic pressure experienced within the first two years, also reflected in our faculty’s 6-year study program’s lowest pass rates. Due to an overwhelming time schedule, studying, and social challenges, it is possible that the students pay less attention to healthy behaviors in this period of their education. However, published data on age and years of study as predictors of health awareness and engagement in healthy behaviors seem to be quite inconsistent and contradictory. Similar to our results, several studies in various settings reported lower HPLP II scores in younger students and those early in their academic education [28,29,30,31]. Conversely, studies on Turkish and Japanese student populations reported a negative correlation between HPLP II scores and the year of university study [19,21].

Overall, students scored highest in the subscales of nutrition, spiritual growth, and particularly interpersonal relationships. Subcategories pertaining to the psychosocial value category (spiritual growth and interpersonal relations) were also scored highest among medical students in Turkey and Croatia and university students in Japan [19,21]. Cultural differences between European and Asian students have already been reported regarding their attitudes towards oral health behavior [32]. Interestingly, it seems that these culturally-based differences cannot be observed among students in relation to their health-promoting lifestyle. The highest scores in the subcategory of interpersonal relationships among Croatian dental students could be attributed to being cultural-specific. Croatians are generally considered to be sociable and to frequently interact with both their primary (i.e., family) and secondary (i.e., friends) spheres of sociability, as reported by a study on sociability patterns [33]. 

Surprisingly, the students scored lowest in the subcategory of health responsibility, which is similar to their Turkish, Iranian, and Japanese colleagues [20,21]. As initially described by Walker et al. [18], this subcategory assesses whether the individual is paying attention and taking responsibility for their health, being educated about health, and seeking timely help when necessary. Individuals at that age are generally of good health and may not perceive it to be necessary to pay much attention to health responsibility. As shown by the multiple regression analysis in our study, a higher BMI seemed to be a predictor of lower health responsibility. However, as reported by Harrington et al., BMI may not be a definite predictor of health behavior. Their findings suggest that both obese and overweight students, as well as students of a healthy weight, may be at risk for poor health behaviors, such as inadequate nutrition and physical inactivity [34]. 

In this study, physical activity was among the overall lowest scored subcategories. When assessing the data in relation to the year of study, it seems that sixth-year students engaged in physical activities the most, as they scored highest in this subcategory. As suggested by the published data, sedentary lifestyle and physical inactivity seem to be a source of concern among college students. A meta-analysis by Keating et al. reported that 40% to 50% of college students are physically inactive [12]. A significant decrease in physical activity seems to follow college enrollment, with time restriction among the most frequently reported reasons for this phenomenon [35]. Croatian population-based studies have highlighted that the lowest physical activity is reported in the 15–24 age group [36], and that 28% of university students are insufficiently physically active [37]. Similar findings on low physical activity scores were also reported by Wei et al. and Lee at al. [21,38]. While in Japan, physical education is not a compulsory course at most universities [21], in Croatia, it is, albeit mostly only in the first year of academic studies. Based on the evidence, it seems that through the university curriculum and development of extracurricular programs, there should be significantly more promotion of physical activity among students.

The results of multiple regression analysis highlighted interesting data on the predictors of stress management. Students of higher year of study and male gender seem to be correlated with better management of stress. The latter was also observed among Croatian medical students [25]. As reported by the dental literature, dental students experience a considerable amount of stress during their education [39,40], more so than their medical counterparts [41]. Stress management training proved to be beneficial when provided to different student populations [42,43], and as such, the implementation of stress management training is warranted in the dental curriculum. In our study, other demographic data, such as smoking and place of residence, did not seem to be good predictors of health-promoting behaviors. Although our multiple regression analysis showed statistically significant correlations between several predictors of a healthy lifestyle and observed dependent variables, the results should be interpreted with caution because of the high-variability data according to the low *R*-squared values (0.05–0.08). This clearly indicates that there are many other factors to consider and explore in future research that definitely influence health-promoting lifestyles for our participants.

The present study has certain limitations. The study was of cross-sectional design, hence reporting data at a specific point in time and not allowing for the analysis of potential changes in the health-promoting behaviors of the studied student population over time. Furthermore, there was a significant difference between the number of students of male and female gender participating in the study, though expected due to the continuous feminization of the dental profession [44,45]. Thus, data regarding differences between the genders should be interpreted with caution. The impact of uncontrolled socially desirable response bias, as often reported in questionnaire-type studies, should also be taken into consideration [46].

At present, the formal education of students on health-promoting behaviors at our faculty is not well defined. Only recently, Zagreb University developed a preventive–promotional framework, educating and supporting students in a healthy lifestyle. Some universities and campuses in the UK [47] and the US [48] have already recognized the importance of health and wellbeing-building environments. As highlighted by Holt et al. [49], these initiatives should be planned and organized to meet the specific needs of their students. This study, along with the data previously published by one of the co-authors [32], provides valuable information on the health-behavior of our faculty’s students, and this information is being used to construct a new health-promoting elective course that is tailored to addressing their particular requirements.

Future research should focus on the assessment of changes in measures of health-promoting lifestyles in the same observed population over a period of time, and more importantly, following a health-promoting intervention. As biomedical students are seen as future health-lifestyle promoters, a significant number of studies are conducted on these populations. However, data on student populations that may not be traditionally associated with health promotion, such as technical faculties, would be of high value. As previously highlighted, tailored health-promoting courses and programs could thus be built around the needs of a specific student population.

## 5. Conclusions

In conclusion, this study highlights that a moderate health-promoting lifestyle can be observed among dental students in Zagreb, Croatia, even in the absence of an official health-promoting educational framework. While students of biomedical faculties exhibit overall favorable health-promoting behaviors, differences among the subcategories may be attributed to specific predictors that have yet to be fully elucidated among different countries and cultures. The development and implementation of promotive and preventive health programs is highly warranted.

## Figures and Tables

**Table 1:** Response rate and distribution of students by academic year, gender, and age.

Academic Year	Total Number *N* (%)	Male *N* (%)	Female *N* (%)	Participating Students *N* (%)	Participants’ Mean Age ± *SD*
Year 1	74 (21.2%)	11 (14.9%)	63 (85.1%)	74 (71.8%)	19.1 ± 0.6
Year 2	52 (14.9%)	11 (21.2%)	41 (78.8%)	52 (55.3%)	20.3 ± 0.7
Year 3	54 (15.5%)	10 (18.5%)	44 (81.5%)	54 (58.1%)	22.7 ± 0.7
Year 4	56 (16.0%)	7 (12.5%)	49 (87.5%)	56 (59.6%)	22.5 ± 1.3
Year 5	57 (16.3%)	4 (7.0%)	53 (93.0%)	57 (54.8%)	23.5 ± 0.9
Year 6	56 (16.0%)	8 (14.3%)	48 (85.7%)	56 (62.2%)	24.5 ± 0.9
Total	349 (100%)	51 (14.6%)	298 (85.4%)	349 (60.3%)	22.3 ± 10.3

*SD*: standard deviation.

**Table 2:** Place of residence and smoking status by academic year.

		Academic Year						
		1	2	3	4	5	6	Total
		*N* (%)	*N* (%)	*N* (%)	*N* (%)	*N* (%)	*N* (%)	*N* (%)
Place of Residence	Family Home	28 (37.8%)	22 (42.3%)	20 (37.0%)	21 (37.5%)	17 (29.8%)	21 (37.5%)	129 (37.0%)
	Dorm	15 (20.3%)	11 (21.2%)	12 (22.2%)	12 (21.4%)	4 (7.0%)	5 (8.9%)	59 (16.9%)
	Rented Apartment	22 (29.7%)	11 (21.2%)	11 (20.4%)	15 (26.8%)	24 (42.1%)	16 (28.6%)	99 (28.4%)
	Other	9 (12.2%)	8 (15.4%)	11 (20.4%)	8 (14.3%)	12 (21.1%)	14 (25.0%)	62 (17.8%)
Smoking	Yes	7 (9.5%)	9 (17.3%)	5 (9.3%)	7 (12.5%)	12 (21.1%)	7 (12.5%)	47 (13.5%)
	No	65 (87.8%)	38 (73.1%)	45 (83.3%)	46 (82.1%)	42 (73.7%)	45 (80.4%)	281 (80.5%)
	Former	2 (2.7%)	5 (9.6%)	4 (7.4%)	3 (5.4%)	3 (5.3%)	4 (7.1%)	21 (6.0%)

**Table 3:** Health-Promoting Lifestyle Profile II (HPLP II) scores according to the academic year.

Year	Overall HPLP II	Health Responsibility	Physical Activity	Nutrition	Spiritual Growth	Interpersonal Relations	Stress Management
Year 1	2.58 ± 0.31 ^f^	2.17 ± 0.54	2.15 ± 0.59 ^f^	2.63 ± 0.45 ^cdf^	2.89 ± 0.44	3.29 ± 0.35	2.29 ± 0.43 ^ef^
Year 2	2.50 ± 0.33 ^cdef^	2.01 ± 0.50	1.99 ± 0.59 ^def^	2.57 ± 0.37 ^cdf^	2.89 ± 0.51	3.26 ± 0.47	2.17 ± 0.42 ^cdef^
Year 3	2.65 ± 0.34 ^b^	2.11 ± 0.71	2.22 ± 0.66 ^f^	2.78 ± 0.36 ^ab^	2.99 ± 0.38	3.33 ± 0.33	2.38 ± 0.42 ^b^
Year 4	2.66 ± 0.38 ^b^	2.22 ± 0.61	2.28 ± 0.78 ^b^	2.79 ± 0.34 ^ab^	2.90 ± 0.48	3.27 ± 0.45	2.41 ± 0.55 ^b^
Year 5	2.67 ± 0.33 ^b^	2.27 ± 0.51	2.30 ± 0.52 ^b^	2.72 ± 0.40 ^f^	2.96 ± 0.51	3.27 ± 0.42	2.46 ± 0.46 ^ab^
Year 6	2.77 ± 0.32 ^ab^	2.22 ± 0.53	2.46 ± 0.66 ^abc^	2.85 ± 0.46 ^ab^	3.06 ± 0.50	3.42 ± 0.31	2.53 ± 0.41 ^abde^
All Students	2.64 ± 0.34	2.17 ± 0.57	2.23 ± 0.65	2.72 ± 0.41	2.95 ± 0.47	3.30 ± 0.39	2.37 ± 0.46
*p* Value	0.001 *	0.216	0.006 *	0.002 *	0.308	0.221	0.001 *

The values are expressed as means ± *SD*; * One-way ANOVA test was conducted, and *p* < 0.05 values are bold; Post hoc tests were done between the years of study: ^a^ Difference is statistically significant from Year 1 (*p* < 0.05). ^b^ Difference is statistically significant from Year 2 (*p* < 0.05). ^c^ Difference is statistically significant from Year 3 (*p* < 0.05). ^d^ Difference is statistically significant from Year 4 (*p* < 0.05). ^e^ Difference is statistically significant from Year 5 (*p* < 0.05). ^f^ Difference is statistically significant from Year 6 (*p* < 0.05).

**Table 4:** Multiple regression analysis of predictors for the overall HPLP II score and certain HPLP II subcategories.

		Unstandardized Coefficients		Standardized Coefficients			
Dependent Variable	Predictor	B	Std. Error	Beta	*t* Value	*p* Value	*R* ^2^
Health Responsibility	Intercept	2.634	0.410		6.423	0.000	0.05
	BMI	−0.033	0.013	−0.149	−2.638	0.009	
	Academic year	0.035	0.018	0.108	1.975	0.049	
Nutrition	Intercept	1.935	0.294		6.576	0.000	0.07
	BMI	0.020	0.009	0.124	2.201	0.028	
	Academic year	0.041	0.013	0.177	3.261	0.001	
Spiritual Growth	Intercept	2.798	0.336		8.323	0.000	0.05
	Age	0.005	0.002	0.113	2.066	0.040	
	Female gender	−0.182	0.075	−0.137	−2.417	0.016	
Stress Management	Intercept	2.393	0.324		7.384	0.000	0.08
	Female gender	−0.224	0.073	−0.171	−3.082	0.002	
	Academic year	0.057	0.014	0.218	4.077	<0.001	
Overall HPLP II Score	Intercept	2.527	0.244		10.343	0.000	0.05
	Academic year	0.040	0.011	0.207	3.800	<0.001	

Note: Only statistically significant (*p* < 0.05) predictors from the regression analysis are shown. BMI: body mass index.

## Data Availability

Not applicable.
